# Fekete-Szegö Inequalities for Certain Classes of Biunivalent Functions

**DOI:** 10.1155/2014/327962

**Published:** 2014-11-06

**Authors:** Şahsene Altınkaya, Sibel Yalçın

**Affiliations:** Department of Mathematics, Faculty of Arts and Science, Uludag University, Bursa, Turkey

## Abstract

We obtain the Fekete-Szegö inequalities for the classes *S*_*S*,Σ_^*^(*α*, *ϕ*) and *£*_*S*,Σ_(*α*, *ϕ*) of biunivalent functions denoted by subordination. The results presented in this paper improve the recent work of Crisan (2013).

## 1. Introduction and Definitions

Let *A* denote the class of analytic functions in the unit disk

(1) $$\begin{array}{c} U=\left\{z\in C:\left|z\right|<1\right\} \end{array}$$

that have the form

(2) $$\begin{array}{c} f\left(z\right)=z+\overset{\infty }{\underset{n=2}{\sum }}a_{n}z^{n}. \end{array}$$

Further, by *S* we will denote the class of all functions in *A* which are univalent in *U*.

The Koebe one-quarter theorem [1] states that the image of *U* under every function *f* from *S* contains a disk of radius 1/4. Thus every such univalent function has an inverse *f* ^−1^ which satisfies

(3) $$\begin{array}{c} f^{-1}\left(f\left(z\right)\right)=z,\;\left(z\in U\right),\\ f\left(f^{-1}\left(w\right)\right)=w,\;\;\left(\left|w\right|<r_{0}\left(f\right),\;r_{0}\left(f\right)\geq \frac{1}{4}\right), \end{array}$$

where

(4) f−1w=w−a2w2+2a22−a3w3 −5a23−5a2a3+a4w4+⋯.

A function $\begin{array}{c} f\left(z\right)\in A \end{array}$ is said to be biunivalent in *U* if both $\begin{array}{c} f\left(z\right) \end{array}$ and $\begin{array}{c} f^{-1}\left(z\right) \end{array}$ are univalent in *U*. Let Σ denote the class of biunivalent functions defined in the unit disk *U*.

If the functions *f* and *g* are analytic in *U*, then *f* is said to be subordinate to *g*, written as

(5) $$\begin{array}{c} f\left(z\right)≺g\left(z\right),\;\left(z\in U\right) \end{array}$$

if there exists a Schwarz function *w*(*z*), analytic in *U*, with

(6) $$\begin{array}{c} w\left(0\right)=0,\;\;\left|w\left(z\right)\right|<1,\;\left(z\in U\right) \end{array}$$

such that

(7) $$\begin{array}{c} f\left(z\right)=g\left(w\left(z\right)\right),\;\left(z\in U\right). \end{array}$$

Lewin [2] studied the class of biunivalent functions, obtaining the bound 1.51 for modulus of the second coefficient |*a*_2_|. Subsequently, Brannan and Clunie [3] conjectured that $\left|a_{2}\right|\leq \sqrt{2}$ for *f* ∈ Σ. Netanyahu [4] showed that max⁡|*a*_2_| = 4/3 if *f*(*z*) ∈ Σ. Brannan and Taha [5] introduced certain subclasses of the biunivalent function class Σ similar to the familiar subclasses of univalent functions consisting of strongly starlike, starlike, and convex functions. They introduced bistarlike functions and obtained estimates on the initial coefficients. Bounds for the initial coefficients of several classes of functions were also investigated in [6–12]. The coefficient estimate problem for each of the following Taylor-Maclaurin coefficients |*a*_*n*_| for *n* ∈ *ℕ*∖{1,2}; *ℕ* = {1,2, 3,…} is presumably still an open problem.

Let *ϕ* be an analytic and univalent function with positive real part in *U* with *ϕ*(0) = 1, *ϕ*′(0) > 0, and *ϕ* maps the unit disk *U* onto a region starlike with respect to 1 and symmetric with respect to the real axis. Taylor's series expansion of such function is of the form

(8) $$\begin{array}{c} \phi \left(z\right)=1+B_{1}z+B_{2}z^{2}+B_{3}z^{3}+\cdots , \end{array}$$

where all coefficients are real and *B*_1_ > 0.

By *S* ^*^(*ϕ*) and *C*(*ϕ*) we denote the following classes of functions:

(9) $$\begin{array}{c} S^{*}\left(\phi \right)=\left\{f:f\in A,\frac{zf^{\prime }\left(z\right)}{f\left(z\right)}≺\phi \left(z\right);\;z\in U\right\},\\ C\left(\phi \right)=\left\{f:f\in A,1+\frac{zf^{\prime \prime }\left(z\right)}{f^{\prime }\left(z\right)}≺\phi \left(z\right);\;z\in U\right\}. \end{array}$$

The classes *S* ^*^(*ϕ*) and *C*(*ϕ*) are the extensions of classical sets of starlike and convex functions and in such a form were defined and studied by Ma and Minda [13]. They investigated growth and distortion properties of functions in *S* ^*^(*ϕ*) and *C*(*ϕ*) as well as Fekete-Szegö inequalities for *S* ^*^(*ϕ*) and *C*(*ϕ*). Their proof of Fekete-Szegö inequalities requires the univalence of *ϕ*. Ali et al. [14] have investigated Fekete-Szegö problems for various other classes and their proof does not require the univalence or starlikeness of *ϕ*. So in this paper, we assume that *ϕ* has series expansion *ϕ*(*z*) = 1 + *B*_1_ *z* + *B*_2_ *z* ^2^ + ⋯, *B*_1_, *B*_2_ are real, and *B*_1_ > 0. A function *f* is bistarlike of Ma-Minda type or biconvex of Ma-Minda type if both *f* and *f* ^−1^ are, respectively, Ma-Minda starlike or convex. These classes are denoted, respectively, by *S*_Σ_^*^(*ϕ*) and *C*_Σ_(*ϕ*) (see [15]).

In [16], Sakaguchi introduced the class *S*_S_^*^ of starlike functions with respect to symmetric points in *U*, consisting of functions *f* ∈ *A* that satisfy the condition *Re*(*zf*′(*z*)/(*f*(*z*) − *f*(−*z*))) > 0, *z* ∈ *U*. Similarly, in [17], Wang et al. introduced the class *C*_*S*_ of convex functions with respect to symmetric points in *U*, consisting of functions *f* ∈ *A* that satisfy the condition *Re*((*zf*′(*z*))′/(*f*′(*z*) + *f*′(−*z*))) > 0, *z* ∈ *U*. In the style of Ma and Minda, Ravichandran (see [18]) defined the classes *S*_S_^*^(*ϕ*) and *C*_*S*_(*ϕ*).

A function *f* ∈ *A* is in the class *S*_S_^*^(*ϕ*) if

(10) $$\begin{array}{c} \frac{2zf^{\prime }\left(z\right)}{f\left(z\right)-f\left(-z\right)}≺\phi \left(z\right),\;z\in U, \end{array}$$

and in the class *C*_*S*_(*ϕ*) if

(11) $$\begin{array}{c} \frac{2{\left(zf^{\prime }\left(z\right)\right)}^{\prime }}{f^{\prime }\left(z\right)+f^{\prime }\left(-z\right)}≺\phi \left(z\right),\;z\in U. \end{array}$$

In this paper, motivated by the earlier work of Zaprawa [19], we obtain the Fekete-Szegö inequalities for the classes *S*_*S*,Σ_^*^(*α*, *ϕ*) and *£*_*S*,Σ_(*α*, *ϕ*). These inequalities will result in bounds of the third coefficient which are, in some cases, better than these obtained in [7].

In order to derive our main results, we require the following lemma.

**Lemma 1 (see [20]).**

If *p*(*z*) = 1 + *p*_1_ *z* + *p*_2_ *z* ^2^ + *p*_3_ *z* ^3^ + ⋯ is an analytic function in *U* with positive real part, then

(12) $$\begin{array}{c} \left|p_{n}\right|\leq 2,\;\left(n\in N=\left\{1,2,\ldots \right\}\right),\\ \left|p_{2}-\frac{p_{1}^{2}}{2}\right|\leq 2-\frac{{\left|p_{2}\right|}^{2}}{2}. \end{array}$$

## 2. Fekete-Szegö Inequalities for the Function Class *S*_*S*,Σ_^*^(*α*, *ϕ*)

**Definition 2 (see [7]).**

A function *f* ∈ Σ is said to be in the class *S*_*S*,Σ_^*^(*α*, *ϕ*) if the following subordination holds:

(13) $$\begin{array}{c} \frac{2\left[\left(1-\alpha \right)zf^{\prime }\left(z\right)+{\alpha z\left(zf^{\prime }\left(z\right)\right)}^{\prime }\right]}{\left(1-\alpha \right)\left(f\left(z\right)-f\left(-z\right)\right)+\alpha z\left(f^{\prime }\left(z\right)+f^{\prime }\left(-z\right)\right)}≺\phi \left(z\right),\\ \frac{2\left[\left(1-\alpha \right)wg^{\prime }\left(w\right)+{\alpha w\left(wg^{\prime }\left(w\right)\right)}^{\prime }\right]}{\left(1-\alpha \right)\left(g\left(w\right)-g\left(-w\right)\right)+\alpha w\left(g^{\prime }\left(w\right)+g^{\prime }\left(-w\right)\right)}≺\phi \left(w\right), \end{array}$$

where *g*(*w*) = *f* ^−1^(*w*).

We note that, for *α* = 0, the class *S*_*S*,Σ_^*^(*α*, *ϕ*) reduces to the class *S*_S_^*^(*ϕ*) introduced by Ravichandran [18].

**Theorem 3 .**

Let *f* given by (2) be in the class *S*_*S*,Σ_^*^(*α*, *ϕ*) and *μ* ∈ *ℝ*. Then

(14) a3−μa22 ≤B121+2α; μ−1  ≤11+2α1+2α+21+α2B1−B2B121−μB1321+2αB12+41+α2B1−B2; μ−1  ≥11+2α1+2α+21+α2B1−B2B12.

Let *f* ∈ *S*_*S*,Σ_^*^(*α*, *ϕ*) and *g* be the analytic extension of *f* ^−1^ to *U*. Then there exist two functions *u* and *v*, analytic in *U* with *u*(0) = *v*(0) = 0, |*u*(*z*)| < 1, |*v*(*w*)| < 1, *z*, *w* ∈ *U*, such that

(15) 2zf′z+αz2f′′z1−αfz−f−z+αzf′z+f′−z=ϕuz,  2zfz+αz2f′′z1−αfz−f−z+αf′z+f′−zz∈U,2wg′w+αw2g′′w1−αgw−g−w+αwg′w+g′−w   =ϕvw, w∈U.

Next, define the functions *p* and *q* by

(16) $$\begin{array}{c} p\left(z\right)=\frac{1+u\left(z\right)}{1-u\left(z\right)}=1+p_{1}z+p_{2}z^{2}+\cdots ,\\ q\left(w\right)=\frac{1+v\left(w\right)}{1-v\left(w\right)}=1+q_{1}w+q_{2}w^{2}+\cdots . \end{array}$$

Clearly, *Rep*(*z*) > 0 and *Req*(*w*) > 0. From (16) one can derive

(17) $$\begin{array}{c} u\left(z\right)=\frac{p\left(z\right)-1}{p\left(z\right)+1}=\frac{1}{2}p_{1}z+\frac{1}{2}\left(p_{2}-\frac{1}{2}p_{1}^{2}\right)z^{2}+\cdots ,\\ v\left(w\right)=\frac{q\left(w\right)-1}{q\left(w\right)+1}=\frac{1}{2}q_{1}w+\frac{1}{2}\left(q_{2}-\frac{1}{2}q_{1}^{2}\right)w^{2}+\cdots . \end{array}$$

Combining (8), (15), and (17),

(18) 2zf′z+αz2f′′z1−αfz−f−z+αzf′z+f′−z =1+12B1p1z+14B2p12+12B1p2−12p12z2+⋯,2wg′w+αw2g′′w1−αgw−g−w+αwg′w+g′−w =1+12B1q1w+14B2q12+12B1q2−12q12w2+⋯.

From (18), we deduce

(19) $$\begin{array}{c} 2\left(1+\alpha \right)a_{2}=\frac{1}{2}B_{1}p_{1}, \end{array}$$

(20) $$\begin{array}{c} 2\left(1+2\alpha \right)a_{3}=\frac{1}{4}B_{2}p_{1}^{2}+\frac{1}{2}B_{1}\left(p_{2}-\frac{1}{2}p_{1}^{2}\right), \end{array}$$

and

(21) $$\begin{array}{c} -2\left(1+\alpha \right)a_{2}=\frac{1}{2}B_{1}q_{1}, \end{array}$$

(22) $$\begin{array}{c} 2\left(1+2\alpha \right)\left(2a_{2}^{2}-a_{3}\right)=\frac{1}{4}B_{2}q_{1}^{2}+\frac{1}{2}B_{1}\left(q_{2}-\frac{1}{2}q_{1}^{2}\right). \end{array}$$

From (19) and (21) we obtain

(23) $$\begin{array}{c} p_{1}=-q_{1}. \end{array}$$

Subtracting (20) from (22) and applying (23) we have

(24) $$\begin{array}{c} a_{3}=a_{2}^{2}+\frac{1}{8\left(1+2\alpha \right)}B_{1}\left(p_{2}-q_{2}\right). \end{array}$$

By adding (20) to (22), we get

(25) $$\begin{array}{c} 4\left(1+2\alpha \right)a_{2}^{2}=\frac{1}{2}B_{1}\left(p_{2}+q_{2}\right)-\frac{1}{4}\left(B_{1}-B_{2}\right)\left(p_{1}^{2}+q_{1}^{2}\right). \end{array}$$

Combining this with (19) and (21) leads to

(26) $$\begin{array}{c} a_{2}^{2}=\frac{B_{1}^{3}\left(p_{2}+q_{2}\right)}{8\left[\left(1+2\alpha \right)B_{1}^{2}+2{\left(1+\alpha \right)}^{2}\left(B_{1}-B_{2}\right)\right]}. \end{array}$$

From (24) and (26) it follows that

(27) a3−μa22=B1hμ+181+2αp2==========+hμ−181+2αq2,

where

(28) $$\begin{array}{c} h\left(\mu \right)=\frac{B_{1}^{2}\left(1-\mu \right)}{8\left[\left(1+2\alpha \right)B_{1}^{2}+2{\left(1+\alpha \right)}^{2}\left(B_{1}-B_{2}\right)\right]}. \end{array}$$

Then, in view of (8) and (12), we conclude that

(29) $$\begin{array}{c} \left|a_{3}-\mu a_{2}^{2}\right|\leq \left\{\begin{array}{cc} \frac{B_{1}}{2\left(1+2\alpha \right)}, & 0\leq \left|h\left(\mu \right)\right|\leq \frac{1}{8\left(1+2\alpha \right)},\\ 4B_{1}\left|h\left(\mu \right)\right|, & \left|h\left(\mu \right)\right|\geq \frac{1}{8\left(1+2\alpha \right)}. \end{array}\right. \end{array}$$

Taking *μ* = 1 or *μ* = 0 we get the following.

**Corollary 4 .**

If *f* ∈ *S*_*S*,Σ_^*^(*α*, *ϕ*) then

(30) $$\begin{array}{c} \left|a_{3}-a_{2}^{2}\right|\leq \frac{B_{1}}{2\left(1+2\alpha \right)}. \end{array}$$

**Corollary 5 .**

If *f* ∈ *S*_*S*,Σ_^*^(*α*, *ϕ*) then

(31) a3≤B121+2α;  B1−B2B12∈−∞,−1+2α1+α2∪0,∞B1321+2αB12+41+α2B1−B2;  B1−B2B12∈−1+2α1+α2,−1+2α21+α2  +++++++∪−1+2α21+α2,0.

**Corollary 6 .**

If

(32) $$\begin{array}{c} \phi \left(z\right)={\left(\frac{1+z}{1-z}\right)}^{\beta }=1+2\beta z+2\beta^{2}z^{2}+\cdots ,\;\left(0<\beta \leq 1\right), \end{array}$$

then inequalities (30) and (31) become

(33) $$\begin{array}{c} \left|a_{3}-a_{2}^{2}\right|\leq \frac{\beta }{1+2\alpha },\;\;\left|a_{3}\right|\leq \frac{\beta }{1+2\alpha }. \end{array}$$

**Corollary 7 .**

If

(34) ϕz=1+1−2βz1−z=1+21−βz+21−βz2+⋯, 0≤β<1,

then inequalities (30) and (31) become

(35) $$\begin{array}{c} \left|a_{3}-a_{2}^{2}\right|\leq \frac{1-\beta }{1+2\alpha },\;\;\left|a_{3}\right|\leq \frac{1-\beta }{1+2\alpha }. \end{array}$$

**Remark 8 .**

Corollaries 6 and 7 provide an improvement of the estimate |*a*_3_| obtained by Crisan [7].

## 3. Fekete-Szegö Inequalities for the Function Class *£*_*S*,Σ_(*α*, *ϕ*)

**Definition 9 (see [7]).**

A function *f* ∈ Σ is said to be *£*_*S*,Σ_(*α*, *ϕ*) if the following subordination holds:

(36) $$\begin{array}{c} {\left(\frac{2zf^{\prime }\left(z\right)}{f\left(z\right)-f\left(-z\right)}\right)}^{\alpha }{\left(\frac{2{\left(zf^{\prime }\left(z\right)\right)}^{\prime }}{f^{\prime }\left(z\right)+f^{\prime }\left(-z\right)}\right)}^{1-\alpha }≺\phi \left(z\right),\\ {\left(\frac{2wg^{\prime }\left(w\right)}{g\left(w\right)-g\left(-w\right)}\right)}^{\alpha }{\left(\frac{2{\left(wg^{\prime }\left(w\right)\right)}^{\prime }}{g^{\prime }\left(w\right)+g^{\prime }\left(-w\right)}\right)}^{1-\alpha }≺\phi \left(w\right), \end{array}$$

where *g*(*w*) = *f* ^−1^(*w*).

We note that, for *α* = 0, the class *£*_*S*,Σ_(*α*, *ϕ*) reduces to the class *C*_*S*_(*ϕ*) introduced by Ravichandran [18].

**Theorem 10 .**

Let *f* given by (2) be in the class *£*_*S*,Σ_(*α*, *ϕ*) and *μ* ∈ *ℝ*. Then

(37) a3−μa22 ≤B123−2α; μ−1≤13−2α +++++×3−3α+α2+22−α2B1−B2B121−μB1323−3α+α2B12+42−α2B1−B2; μ−1≥13−2α +++++×3−3α+α2+22−α2B1−B2B12.

Let *f* ∈ *£*_*S*,Σ_(*α*, *ϕ*) and *g* be the analytic extension of *f* ^−1^ to *U*. Then there exist two functions *u* and *v*, analytic in *U* with *u*(0) = *v*(0) = 0, |*u*(*z*)| < 1, |*v*(*w*)| < 1, *z*, *w* ∈ *U*, such that

(38) $$\begin{array}{c} \frac{2{\left(zf^{\prime }\left(z\right)\right)}^{\prime }}{f^{\prime }\left(z\right)+f^{\prime }\left(-z\right)}=\phi \left(u\left(z\right)\right),\\ \frac{2{\left(wg^{\prime }\left(w\right)\right)}^{\prime }}{g^{\prime }\left(w\right)+g^{\prime }\left(-w\right)}=\phi \left(v\left(w\right)\right). \end{array}$$

From (38), we deduce

(39) $$\begin{array}{c} 2\left(2-\alpha \right)a_{2}=\frac{1}{2}B_{1}p_{1}, \end{array}$$

(40) $$\begin{array}{c} 2\left(3-2\alpha \right)a_{3}-2\alpha \left(1-\alpha \right)a_{2}^{2}=\frac{1}{4}B_{2}p_{1}^{2}+\frac{1}{2}B_{1}\left(p_{2}-\frac{1}{2}p_{1}^{2}\right), \end{array}$$

and

(41) $$\begin{array}{c} -2\left(2-\alpha \right)a_{2}=\frac{1}{2}B_{1}q_{1}, \end{array}$$

(42) 23−2α2a22−a3−2α1−αa22 =14B2q12+12B1q2−12q12.

From (39) and (41) we obtain

(43) $$\begin{array}{c} p_{1}=-q_{1}. \end{array}$$

Subtracting (40) from (42) and applying (43) we have

(44) $$\begin{array}{c} a_{3}=a_{2}^{2}+\frac{1}{8\left(3-2\alpha \right)}B_{1}\left(p_{2}-q_{2}\right). \end{array}$$

By adding (40) to (42), we get

(45) $$\begin{array}{c} 4\left(3-3\alpha +\alpha^{2}\right)a_{2}^{2}=\frac{1}{2}B_{1}\left(p_{2}+q_{2}\right)-\frac{1}{4}\left(B_{1}-B_{2}\right)\left(p_{1}^{2}+q_{1}^{2}\right). \end{array}$$

Combining this with (39) and (41) leads to

(46) $$\begin{array}{c} a_{2}^{2}=\frac{B_{1}^{3}\left(p_{2}+q_{2}\right)}{8\left[\left(3-3\alpha +\alpha^{2}\right)B_{1}^{2}+2{\left(2-\alpha \right)}^{2}\left(B_{1}-B_{2}\right)\right]}. \end{array}$$

From (44) and (46) it follows that

(47) a3−μa22=B1hμ+183−2αp2+++++++++hμ−183−2αq2,

where

(48) $$\begin{array}{c} h\left(\mu \right)=\frac{B_{1}^{2}\left(1-\mu \right)}{8\left[\left(3-3\alpha +\alpha^{2}\right)B_{1}^{2}+2{\left(2-\alpha \right)}^{2}\left(B_{1}-B_{2}\right)\right]}. \end{array}$$

Then, in view of (8) and (12), we conclude that

(49) a3−μa22≤B123−2α,0≤hμ≤183−2α,4B1hμ,hμ≥183−2α.

Taking *μ* = 1 or *μ* = 0 we get the following.

**Corollary 11 .**

If *f* ∈ *£*_*S*,Σ_(*α*, *ϕ*) then

(50) $$\begin{array}{c} \left|a_{3}-a_{2}^{2}\right|\leq \frac{B_{1}}{2\left(3-2\alpha \right)}. \end{array}$$

**Corollary 12 .**

If *f* ∈ *£*_*S*,Σ_(*α*, *ϕ*) then

(51) a3≤B123−2α; B1−B2B12∈−∞,α−322−α++++++++∪α1−α22−α2,∞B1323−3α+α2B12+42−α2B1−B2; B1−B2B12∈α−322−α,3α−α2−322−α2 +++++++∪3α−α2−322−α2,α1−α22−α2.

**Corollary 13 .**

If

(52) $$\begin{array}{c} \phi \left(z\right)={\left(\frac{1+z}{1-z}\right)}^{\beta }=1+2\beta z+2\beta^{2}z^{2}+\cdots ,\;\left(0<\beta \leq 1\right), \end{array}$$

then inequalities (50) and (51) become

(53) $$\begin{array}{c} \left|a_{3}-a_{2}^{2}\right|\leq \frac{\beta }{3-2\alpha },\;\;\left|a_{3}\right|\leq \frac{\beta }{3-2\alpha }. \end{array}$$

**Corollary 14 .**

If

(54) ϕz=1+1−2βz1−z=1+21−βz+21−βz2+⋯, 0≤β<1,

then inequalities (50) and (51) become

(55) $$\begin{array}{c} \left|a_{3}-a_{2}^{2}\right|\leq \frac{1-\beta }{3-2\alpha },\;\;\left|a_{3}\right|\leq \frac{1-\beta }{3-3\alpha +\alpha^{2}}. \end{array}$$

**Remark 15 .**

Corollaries 13 and 14 provide an improvement of the estimate |*a*_3_| obtained by Crisan [7].
